# Economic difficulties and subsequent sleep problems: Evidence from British and Finnish occupational cohorts

**DOI:** 10.1016/j.sleep.2011.10.036

**Published:** 2012-06

**Authors:** Tea Lallukka, Jane E. Ferrie, Mika Kivimäki, Martin J. Shipley, Ossi Rahkonen, Eero Lahelma

**Affiliations:** a Hjelt Institute, Department of Public Health, University of Helsinki, Finland; b Department of Epidemiology and Public Health, University College London, London, UK; c School of Community and Social Medicine, University of Bristol, UK

**Keywords:** Financial problems, Follow-up, Insomnia, International, Lifecourse, Socioeconomic

## Abstract

**Background:**

Social determinants of sleep may prove to be as important as health status. In this study we examined the extent to which persistent and changing economic difficulties are associated with sleep problems in two prospective occupational cohorts.

**Methods:**

We used data from Finnish (baseline 2000–2002; follow-up 2007; *n* = 6328) and British (baseline 1997–1999; follow-up 2003–2004; *n* = 5002) public sector employees. Economic difficulties, sleep problems, and a variety of covariates were assessed at baseline and follow-up.

**Results:**

Prevalence of frequent sleep problems at follow-up was 27% and 20% among women and men in the Finnish cohort, and 34% and 27% in the British cohort, respectively. Odds for sleep problems were higher among those with persistent economic difficulties (frequent economic difficulties at baseline and follow-up) compared to those with no difficulties. This association remained after multiple adjustments, including parental and current socioeconomic position, in the Finnish (OR 1.72, 95% CI 1.35–2.18) cohort. Increases in economic difficulties were similarly associated with sleep problems in the Finnish and the British cohort.

**Conclusion:**

Evidence from two occupational cohorts suggests strong associations between economic difficulty and poor sleep. Awareness of this association will help health care professionals identify and prevent sleep problems.

## 1 Introduction

Sleep problems are prevalent and they are associated with subsequent mental and physical health [1–3]. Prospective studies suggest that they result in a substantial cost to society in terms of absenteeism, work disability, occupational injuries, and use of health care [2,4–8]. Sleep problems also tend to be patterned by socioeconomic circumstances, potentially contributing to socioeconomic inequalities in health [9,10]. However, not all studies have found sleep problems to be more common in lower socioeconomic positions, delineated by conventional indicators of socioeconomic position, education, occupational class, or income [9,11–15].

Economic difficulties examined in this study are conceptualised as a separate domain of socioeconomic circumstances that make a contribution to health over and above the effects of conventional indicators. More specifically, they indicate present material hardship in terms of difficulties in the payment of bills and purchase of food or clothing [16–19]. Accordingly, the association of economic difficulties with behavioural risk factors [20,21] and health [17–19] is independent of conventional indicators of socioeconomic position, highlighting the significance of economic difficulties for health. Although economic difficulties tend to be more prevalent among those in lower socioeconomic positions, economic difficulties should not be seen as a proxy for disposable income or lower status only, as economic difficulties can exist at all income levels [22]. Furthermore, they are associated with adverse behaviours such as smoking even among those with a high income [23].

A small number of cross-sectional studies have found economic difficulties, and, more broadly, material circumstances, to be associated with sleep independent of other measures of socioeconomic position, past and present [11,12,24]. However, measures of economic difficulties in existing studies have varied from concrete difficulties [24] to perceived financial strain [12] or economic deprivation [11]. Measures of sleep problems have also varied between studies. In contrast to the cross-sectional evidence, economic difficulties were unassociated with sleep problems in a prospective US cohort of non-institutionalised older people [25]. In all of these studies economic difficulties were measured at one point in time only, and associations between change or persistence of economic difficulties and sleep problems could not be assessed.

Persistent economic difficulties have been shown to predict serious health outcomes, such as incident coronary events [17], but we are unaware of previous longitudinal studies examining their consequences for subsequent sleep problems. Given the indication of an association between economic difficulties and sleep problems in cross-sectional data, we hypothesised that emergent or persistent economic difficulties may be associated with subsequent sleep problems. We also hypothesised that these associations would remain after taking into account baseline sleep problems and multiple indicators of childhood and current socioeconomic position. These hypotheses were tested using repeat measures from Finnish and British prospective occupational cohorts that have the advantage of harmonised key variables.

## 2 Methods

### 2.1 Participants

Public sector employee cohort data were available from Finland and Britain. These data were comparable in terms of data collection period, content of measures, age, and employment status. The Finnish Helsinki Health Study data were derived from baseline (2000–2002) and follow-up (2007) postal surveys among the staff of the City of Helsinki (*n* = 6328) [26]. The response rate was 67% at baseline and 83% at follow-up. At baseline, all participants, aged 40–60, were employed, and 71% continued to be employed over the follow-up. The baseline data broadly represent the target population [27,28]. Practically all participants in the Helsinki Health Study were Finnish, except for a Swedish speaking minority (less than 10%).

The British Whitehall II is a cohort of 10,308 white-collar civil servants drawn from 20 London-based civil service departments and aged 35–55 on entry to the study (1985–1988) [29]. To harmonise age ranges and assessment periods between the two cohorts baseline for the present analysis is phase 5 (1997–1999) and follow-up is phase 7 (2003–2004). All participants who were employed at phase 5 and participated in both phases were included. Corresponding to the Finnish cohort, 72% were employed at follow-up. Most participants in the Whitehall II study are white (92%), but there are also small groups of Afro-Caribbean and Asian participants (Black-Caribbean, Black-African, Indian, Pakistani, Bangladeshi, Chinese).

Ethical approval for the Helsinki Health Study came from the Department of Public Health, University of Helsinki, and the City of Helsinki. Corresponding approval for the Whitehall II study came from the University College London Ethics Committee.

### 2.2 Measures

Questions on economic difficulties covered the purchase of food and clothes (five response categories, range “always” to “never”) and difficulty paying bills (five response categories, range “very little” to “very much”) [16]. Responses were combined to form three categories of economic difficulties: frequent, occasional, and none. Further details of the economic difficulties measure have been reported elsewhere [17–19,24].

Participants were classified into nine categories of change in economic difficulties over time; three of these categories represent no change between baseline and follow-up (none–none, occasional–occasional, frequent–frequent); six of these categories represent change (decrease from occasional to none, from frequent to occasional, and from frequent to none; increase from none to occasional, from occasional to frequent, and from none to frequent).

Sleep problems measured by the Jenkins sleep questionnaire included difficulties with sleep onset, sleep maintenance, and non-restorative sleep at baseline and follow-up [30]. Questions asked whether these problems had occurred during the previous four weeks: (1) not at all, (2) 1–3 days, (3) 4–7 days, (4) 8–14 days, (5) 15–21 days, and (6) 22–28 days. Categories 5 and 6 were collapsed to form frequent sleep problems, as in previous studies [7,31].

Sociodemographic factors, socioeconomic position, and employment status were included as covariates. Childhood economic difficulties (yes/no) referred to serious financial difficulties in the childhood family when the participants were less than 16 years old. Marital status was classified into three groups: single, married or cohabiting, and divorced or widowed.

Own education was categorised into three groups in both cohorts: high (university degree), intermediate, and low education. Three occupational classes were used in the Finnish cohort: low (routine non-manual employees); intermediate (semi-professionals and professionals); and high (managers). In the British cohort, three corresponding categories were used: low (clerical and administrative support staff); intermediate (professional and executive staff); and high (senior administrative staff and managers). Household income was reported after taxes, taking into account any welfare benefits and other sources of income received during an average month in the Finnish cohort, and in the previous 12 months in the British cohort. Household income was weighted by the number of people living in the household [32]. Weighted household income was divided into quartiles of “very low,” “low,” “high,” and “very high.” Cut-off points were sex-specific, since men reported higher income. Similar income data were derived from the follow-up survey.

Housing tenure was classified as owner–occupier and renter/other. Finally, employment status reported at follow-up differentiated between those continuously employed and those retired, unemployed, or otherwise out of the labour market. Further details of these covariates can be found in our previous reports [22,24].

### 2.3 Statistical analyses

Logistic regression analysis was used to examine associations of exposure to economic difficulties at baseline and follow-up with sleep problems at follow-up (odds ratios, OR, and their 95% confidence intervals, CI). As no interactions between sex and the measures of economic difficulties were found, data were pooled and adjusted for sex. Odds ratios were sequentially adjusted for age and sex (Model 0), baseline sleep problems (Model 1), childhood economic difficulties (Model 2), marital status, education, occupational class, household income at baseline and at follow-up, housing tenure, and employment status at follow-up (Model 3). Income and employment status at follow-up were used to take into account changes in income level and exit from workforce after baseline. Housing tenure, available from baseline in both cohorts, was used to take into account material circumstances over a longer time period. Sensitivity analyses adjusted for a measure of wealth (total household assets including the value of the house after paying off any debts and mortgage), available only at follow-up in the Finnish data, was used as a further discriminator of material resources, but the results remained similar (data not shown).

Multiple imputation for missing values was conducted using the aregImpute function in the Hmisc package for R software (R Foundation for Statistical Computing, Vienna). With this function, multiple imputation is based on additive regression, bootstrapping, and predictive mean matching as described elsewhere [33]. During the imputation process, 10 imputed datasets were created, assuming missing at random [33]. All the analyses with the imputed datasets were computed using the R program. Sensitivity analyses and variable construction were conducted using the SAS statistical program, version 9.2.

## 3 Results

Sleep problems were more prevalent among women than men and more prevalent in the British than the Finnish cohort. At baseline, 21% of Finnish women and 31% of British women reported frequent sleep problems. The corresponding figures among men were 16% and 22%. At follow-up, the prevalence of frequent sleep problems was 27% among Finnish women and 34% among British women, respectively, and 20% and 27%, respectively, among men (Table 1).

In contrast, differences in the level of the economic difficulties and their changes were minor between the two cohorts. Frequent economic difficulties at baseline and follow-up were reported by 10% of the Finnish, and by 7% of the British cohort (Table 1). Over the follow-up, 43% of women and 50% of men in the Finnish cohort reported no economic difficulties, while in the British cohort 46% of women and 51% of men reported no economic difficulties at either phase. There was a tendency for economic difficulties to decrease over the follow-up period, with 21% and 24% of participants in the Finnish and British cohorts reporting a decrease in economic difficulties compared with 16% and 10% reporting an increase.

Strong associations were observed between persistent frequent economic difficulties and sleep problems. These remained after adjustment for age, sex, baseline sleep problems, childhood economic difficulties, marital status, education, occupational class, household income at baseline and follow-up, housing tenure, and employment status at follow-up in the Finnish cohort (OR 1.72, 95% CI 1.35–2.18). In the British cohort, the corresponding association was found in the age and sex adjusted model (OR 1.35, 95% CI 1.05–1.73) (Table 2). However, when stricter criteria for classification of persistent frequent economic difficulties were applied (prevalence 2.4%), the association remained strong and similar to the Finnish cohort also in the British cohort throughout the modelling. In the pooled analyses, persistent frequent economic difficulties also remained associated with sleep problems after full adjustments, and no interaction between cohort and economic difficulties was found (data not shown).

An increase in economic difficulties over the follow-up (from “none” at baseline to “frequent” at follow-up) was also associated with sleep problems at follow-up in the Finnish cohort after full-adjustment (OR 1.81, 95% CI 1.22–2.68). In the British cohort, evidence for this association was strong and statistically significant only in the age and sex adjusted model (OR 1.64, 95% CI 1.00–2.69), although the estimates remained equal, in terms of effect size, after full adjustment. An increase in economic difficulties from occasional to frequent was also associated with sleep problems in the Finnish (OR 1.73, 95% CI 1.30–2.32) and the British (OR 1.51, 95% CI 1.00–2.27) cohort, after full adjustments but an increase from none to occasional had no effect. There was no strong evidence in either cohort that decreases in economic difficulties were associated with sleep problems.

## 4 Discussion

This study utilised follow up survey data from Finnish and British occupational cohorts to examine associations of changes in economic difficulties and persistent economic difficulties with subsequent sleep problems. Increasing economic difficulties were consistently associated with sleep problems at follow-up in both cohorts. This association remained after adjustment for a range of covariates, including indicators of socioeconomic position, childhood economic difficulties, and baseline sleep problems. Persistent frequent economic difficulties over the follow-up period were also strongly associated with sleep problems in the Finnish cohort.

### 4.1 Comparison with previous studies

As we lack previous studies on change in and persistence of economic difficulties, comparability of our findings to previous studies remains limited. In line with our study, a few studies have observed associations between economic difficulties, or related material circumstances, and sleep [11,12,24,25,34]. However, these studies have mostly been cross-sectional, have examined heterogeneous study populations, and have used varying measures of economic difficulties and sleep. Contrary to this, one prospective study in an older cohort with a one year follow-up found no association between baseline economic difficulties and subsequent sleep problems at follow-up [25]. However, this study also lacked repeat measurements of economic difficulties and, thus, did not focus on changes in difficulties.

In addition to economic difficulties perceived at the individual level, other context specific or period effects, such as economic downturn, may contribute to sleep problems. Accordingly, some studies have focused on sleep problems during economic downturns or recessions [34,35]. In a British study, reported economic difficulties were associated with sleep problems only during economic downturn, which suggests that it is important to consider potential period effects [34]. However, an earlier study found no evidence of deterioration in sleep quality during a major economic recession in Finland compared to levels of sleep problems before the recession [35]. As an explanation for the null finding the authors suggested that the economic recession affected all population groups and was much more severe in Finland than, for example, Britain, resulting in a dilution of effects when being unemployed was common and unlikely to cause major isolation or psychological stigma [35]. However, it needs to be noted that the study did not focus on economic difficulties. In other words, although an economic recession is not equal to economic difficulties, it can be expected that during recession and periods of high unemployment the prevalence of economic difficulties overall, albeit more concentrated in some population subgroups, is likely to be high.

Childhood economic difficulties and other adversities have previously been associated with adult sleep problems, at least in two studies [24,36]. As sleep problems were more prevalent than economic difficulties, it might be surmised that economic difficulties in adulthood make only a minor contribution to sleep problems, raising the possibility that other factors, such as childhood economic difficulties, play an important role. However, in the present study, childhood economic difficulties made a negligible contribution to the association between economic difficulties and sleep problems in both cohorts. Further, to investigate the lack of contribution of childhood economic difficulties, analyses stratified by level of childhood economic difficulties were conducted combining both cohorts in a pooled dataset (data not shown). These analyses showed similar associations between persistent and increasing difficulties, with sleep problems both among those who did and among those who did not report childhood economic difficulties. Childhood economic difficulties were reported by around one fifth or one quarter of participants, and only partly overlapped with current economic difficulties. These findings suggest that current economic difficulties and their associations with sleep problems do not reflect sensitivity to exposure and childhood adversity.

Further considerations of mechanisms or pathways that might account for or mediate the association between changes in economic difficulties and sleep problems include changes in health status. Our earlier studies have shown economic difficulties to be associated with both physical and mental functioning in the cohorts examined [18,19]. As sleep problems are closely linked to physical and mental health [1], this might account for part of the observed effects. However, it is equally possible that poor sleep serves as a mechanism explaining the link between economic difficulties and ill-health [9,10]. Behavioural and lifestyle-related factors, and changes in them over the follow-up period, could also serve as explanations of the associations. Having data from two time points only limits the possibilities to address these issues and causal order more closely. Other explanations for the associations could involve psychosocial pathways, as economic difficulties are very likely to cause stress that in turn affects sleep [37]. Additionally, work–family conflicts could mediate the association, as they are strongly associated with sleep problems [38], and also contributed to the associations observed between economic difficulties and health-outcomes [18,19]. For example, increasing economic difficulties may be related to changes in work, or lead to efforts to compensate for the situation by increasing working hours, taking another job, or merely worrying about the situation at home, causing conflicts and subsequent sleep problems. Alternatively, economic difficulties may be due to situations outside work, such as family-related or personal issues and problems, and so contribute to psychosocial stress or work–family conflicts, and subsequent sleep problems. However, data on work–family conflicts to further elaborate these issues are only available for some of the participants in this study. More detailed examination of the reasons behind the associations observed is beyond the scope of this study, but warrants further scrutiny. In addition to work–family conflicts, further social and family related factors, such as family composition and living arrangements, and changes in these, could contribute to the observed associations.

Baseline sleep problems mostly had a minor effect on the studied associations. A tenth of the Finnish study population and 14% of the British cohort reported sleep problems at both time points. Among those reporting frequent sleep problems at baseline, 57% and 59% also reported frequent sleep problems at follow-up in the Finnish and British cohorts, respectively. This is in agreement with previous studies suggesting that sleep problems tend to be long-lasting [25,39].

As women in this study, in common with women in general, report more sleep problems and economic difficulties than men [9,18], the unequal sex distribution within these two cohorts could suggest that, in the pooled analyses, results from men dominate the British cohort and those from women dominate the Finnish cohort. We initially conducted sex stratified analyses, but no sex interactions were found. Because of this lack of interaction, and as the numbers of men in the Finnish cohort, and the numbers of women in the British cohort, are relatively low, we preferred to present results from the pooled data, as the models are more stable than those in the sex-stratified analyses.

Although we cannot rule out that our observed associations are explained by differences in individual characteristics, such as ability to budget within ones means, our findings provide strong evidence that economic difficulties have a robust association with sleep that captures aspects not covered by other measures of socioeconomic disadvantage.

### 4.2 Methodological considerations

Several further limitations of this study should be acknowledged. First, a limitation of this study is the generalizability of the findings as a cohort of middle-aged public sector employees is representative of only one sector of the workforce. Second, data collection in the Whitehall II study began some time before that in the Helsinki Health Study. To enable us to avoid varying period effects, and to compare employees of similar ages, we used phase 5 of the Whitehall II study as the baseline. Although phase 5 data were collected 10–12 years after the study baseline, previous analyses suggest selective loss to follow-up or attrition is unlikely to have substantially distorted the data [22]. More specifically, the associations between economic difficulties, other socioeconomic circumstances, and common mental disorders in phases 1 and 3 were broadly similar to the association observed at phase 5, thus showing that the effect of attrition on this association is minor. Data on sleep problems were not collected in the early phases, but the associations are likely to follow patterns similar to the previously examined mental disorders. Moreover, non-response to follow-up was associated with a similar level of excess mortality risk as non-response to baseline [40]. In the Helsinki Health Study the high response to follow-up (83%) suggests that attrition, overall, is unlikely to be highly selective, although we have shown that younger men and those in lower socioeconomic groups were somewhat more likely to drop out compared to other participants (further data not shown). Although some attrition is to be expected in long follow-up surveys, our data have thus remained broadly representative and attrition is unlikely to have distorted the association between economic difficulties and sleep problems. Third, the prevalence of increasing economic difficulties (from none to frequent) was very low in both cohorts. However, as the association was similar to persistent economic difficulties in the Finnish cohort, this suggests that such difficulties are equally important to sleep. Fourth, our classification of economic difficulties into three categories, both at baseline and follow-up, allowed us to examine associations with sleep problems without making any assumptions of linearity of effect across the range of difficulties. In all of our models we fitted interaction terms between baseline and follow-up economic difficulties, which allowed the effects of changes in economic difficulties to differ according to the baseline level and between increasing versus decreasing difficulties. We also examined the associations between economic difficulties and sleep problems separately for low and high income groups (data not shown). The associations were equally strong among both groups, but confidence intervals were wider, limiting the precision of the estimates. Income level, in turn, had weak or practically non-existent independent associations with sleep problems. Cross-sectional analyses between economic difficulties and sleep suggested that frequent economic difficulties, in particular, are associated with sleep problems, whereas the effect was weak or non-existent for occasional sleep problems (data not shown). Fifth, when adjusting for several socioeconomic circumstances, multicollinearity could emerge as a problem. However, although indicators of socioeconomic circumstances correlate, there was no indication of multicollinearity in these data based on low VIF values tested in the regression models including all the socioeconomic indicators simultaneously. This suggests that simultaneous adjustment for all indicators provides precise estimates. While economic difficulties exist at all income levels, they are likely to arise for partly different reasons among low and high income groups. Furthermore, as socioeconomic position is a broad umbrella concept, its effects cannot be captured by one indicator only. To be able to show the independent effect of economic difficulties on sleep, we took into account other indicators of socioeconomic position and material circumstances more broadly. Sixth, it is possible that participants made frequent transitions into and out of economic difficulty during the relatively long follow-up period. There are no data available that document such changes or their timing. It cannot be ruled out that those whose economic difficulties disappeared during follow-up had greater exposure to economic difficulties than those with emerging economic difficulties. Seventh, measurement error is a further potential methodological limitation, as reporting on the level of economic difficulties can be biased. Finally, due to the large number of covariates included, the proportion of missing items increased (around 10–30% altogether), and we thus used imputation to maximise the number of participants in the analyses and to minimise the possibility of selection bias. However, complete case analyses produced similar or even slightly stronger associations (data not shown). We preferred to retain the full sample, i.e., we examined the imputed data.

The main strength of this study was the use of prospective data with identical measurements from two independent cohorts. The data were large and composed of both women and men. As both economic difficulties and sleep problems were measured similarly at baseline and follow-up, we were able to examine changes in economic difficulties. Furthermore, our ability to control for the effects of education, occupational class, and changes in income in the analysis meant that we were able to demonstrate consistent evidence regarding the significance of persistent and increasing economic difficulties for future sleep quality independent of socioeconomic position.

## 5 Conclusions

Persistent and increasing frequent economic difficulties were associated with sleep problems in cohorts from two countries. In the Finnish cohort, these associations were robust to adjustment for other measures of adult socioeconomic position, as well as adjustment for childhood economic difficulties. This suggests that the associations of current economic difficulties with adult sleep are not accounted for by other measures of adult socioeconomic position, childhood disadvantage, or sensitivity to the exposure. While sleep problems are known to be associated with poor physical and mental health, it is important to note that sleep is affected by other factors besides health. These include factors in the physical environment such as noise, health-related behaviours such as alcohol consumption, and conventional socioeconomic circumstances such as socioeconomic position [24,38,41–43]. In our paper we show that material hardship, measured as economic difficulties in everyday life, is also adversely associated with sleep. Our findings are of relevance to health care professionals and GPs who are presented with patients suffering from sleep problems without an apparent health-related cause. While health-care professionals and GPs are not able to directly address their patients’ economic difficulties, they can use their offices to help patients obtain support – for example, from social services. As sleep problems contribute to subsequent ill-health and disability retirement, it is important to try to rectify both the economic difficulties and the sleep problems at an early stage to prevent them from becoming chronic.

## Conflict of Interest

The ICMJE Uniform Disclosure Form for Potential Conflicts of Interest associated with this article can be viewed by clicking on the following link: doi:10.1016/j.sleep.2011.10.036.

Conflicts of Interest ICMJE Form for Disclosure of Potential Conflicts of Interest form.

## Figures and Tables

**Table 1:** Distributions (%) of key study variables in the Finnish Helsinki Health Study and the British Whitehall II occupational cohorts.

	Helsinki Health Study^a^			Whitehall II Study^b^		
	All (n = 6328) %	Women (n = 5304) %	Men (n = 1024) %	All (n = 5002) %	Women (n = 1342) %	Men (n = 3660) %
Sleep problems at baseline	19.8	20.6	15.9	24.4	30.8	22.1

Sleep problems at follow-up	25.7	26.7	20.2	28.6	34.3	26.5

Current economic difficulties						
No change (None-None)	43.7	42.5	50.0	49.9	46.2	51.3
No change (Occasional-Occasional)	9.8	10.0	8.8	9.1	10.3	8.7
No change (Frequent-Frequent)	9.8	10.3	7.1	7.1	9.0	6.4
Decrease (Occasional-None)	10.2	10.0	11.2	13.5	12.5	13.9
Decrease (Frequent-Occasional)	5.7	5.8	4.8	5.4	6.0	5.2
Decrease (Frequent-None)	4.7	4.4	5.8	4.7	4.6	4.8
Increase (None-Occasional)	8.2	8.6	6.4	5.3	5.5	5.2
Increase (Occasional-Frequent)	5.3	5.6	4.0	3.3	3.9	3.1
Increase (None-Frequent)	2.7	2.8	1.9	1.6	2.0	1.5

a Baseline (2000–2002) and follow-up (2007) surveys.

b Baseline (phase 5, 1997–1999), and follow-up (phase 7, 2003–2004), participants who were working at phase 5 and all participants at follow-up.

**Table 2:** Associations between changes in economic difficulties and sleep problems at follow-up. Odds ratios (OR) and their 95% confidence intervals (CI).

Economic difficulties at baseline and follow-up	Model 0: Age adjusted for		Model 1: Model 0+baseline sleep problems		Model 2: Model 1+childhood economic difficulties		Model 3: Model 2+sociodemographic and socioeconomic factors^a^	
	OR	95% CI	OR	95% CI	OR	95% CI	OR	95% CI 9
Helsinki Health Study, Finland (n = 6328)								

*Economic difficulties at baseline and follow-up*								
No change (None-None)	1.00		1.00		1.00		1.00	
No change (Occasional-Occasional)	1.05	(0.85–1.30)	1.09	(0.86–1.36)	1.07	(0.85–1.35)	1.13	(0.89–1.43)
No change (Frequent-Frequent)	1.86	(1.52–2.27)	1.63	(1.32–2.02)	1.59	(1.29–1.97)	1.72	(1.35–2.18)
Decrease (Occasional-None)	1.06	(0.86–1.30)	1.02	(0.81–1.27)	1.00	(0.80–1.26)	1.02	(0.81–1.28)
Decrease (Frequent-Occasional)	1.08	(0.83–1.41)	0.98	(0.74–1.31)	0.97	(0.73–1.29)	1.05	(0.78–1.41)
Decrease (Frequent-None)	1.26	(0.95–1.67)	1.11	(0.81–1.52)	1.09	(0.79–1.49)	1.13	(0.82–1.55)
Increase (None-Occasional)	1.00	(0.79–1.26)	0.94	(0.73–1.20)	0.93	(0.73–1.20)	0.98	(0.76–1.26)
Increase (Occasional-Frequent)	1.64	(1.26–2.13)	1.64	(1.25–2.16)	1.61	(1.22–2.13)	1.73	(1.30–2.32)
Increase (None-Frequent)	1.84	(1.29–2.62)	1.68	(1.15–2.45)	1.66	(1.14–2.43)	1.81	(1.22–2.68)

Whitehall II Study, UK (n = 5002)								

*Economic difficulties at baseline and follow-up*								
No change (None-None)	1.00				1.00		1.00	
No change (Occasional-Occasional)	1.18	(0.92–1.51)	1.08	(0.83–1.42)	1.07	(0.82–1.40)	1.12	(0.84–1.48)
No change (Frequent-Frequent)	1.35	(1.05–1.73)	1.19	(0.91–1.57)	1.16	(0.88–1.53)	1.27	(0.94–1.71)
Decrease (Occasional-None)	1.06	(0.87–1.30)	1.01	(0.81–1.26)	1.01	(0.81–1.26)	1.04	(0.83–1.30)
Decrease (Frequent-Occasional)	1.15	(0.85–1.56)	1.10	(0.80–1.52)	1.08	(0.78–1.50)	1.18	(0.84–1.65)
Decrease (Frequent-None)	1.25	(0.90–1.73)	1.23	(0.87–1.73)	1.21	(0.85–1.70)	1.29	(0.91–1.83)
Increase (None-Occasional)	1.05	(0.76–1.45)	0.96	(0.67–1.38)	0.95	(0.66–1.37)	0.95	(0.66–1.37)
Increase (Occasional-Frequent)	1.46	(1.01–2.13)	1.43	(0.95–2.16)	1.42	(0.94–2.14)	1.51	(1.00–2.27)
Increase (None-Frequent)	1.64	(1.00–2.69)	1.56	(0.91–2.68)	1.54	(0.90–2.65)	1.58	(0.91–2.74)

a Marital status, education, occupational class, household income at baseline and follow-up, housing tenure, employment status at follow-up.
