# Erratum to “Economic difficulties and subsequent sleep problems: Evidence from British and Finnish occupational cohorts” [Sleep Med 12 (6) (2012) 680–685]

**DOI:** 10.1016/j.sleep.2012.08.001

**Published:** 2012-10

**Authors:** Tea Lallukka, Jane E. Ferrie, Mika Kivimäki, Martin J. Shipley, Ossi Rahkonen, Eero Lahelma

**Affiliations:** a Hjelt Institute, Department of Public Health, University of Helsinki, Finland; b Department of Epidemiology and Public Health, University College London, London, UK; c School of Community and Social Medicine, University of Bristol, UK

During the imputation of missing data, a number of additional participants from Whitehall phase 5 were included by mistake. The correct number in the analyses should have been 4350, not 5002. Re-running the analyses excluding these additional participants from the imputation did not affect the results. Thus the associations remained very similar to those reported.

Complete case analysis reported in the ‘Discussion’ was done with the correct number of participants in the first instance. Corrected tables are available from the first author on request.

